# Real-Time Monitoring of *Tetraselmis suecica* in A Saline Environment as Means of Early Water Pollution Detection

**DOI:** 10.3390/toxics6040057

**Published:** 2018-09-28

**Authors:** Karin Brenda Moejes, Reshma Sulthana Rahiman Sherif, Simone Dürr, Sheelagh Conlan, Alex Mason, Olga Korostynska

**Affiliations:** 1 Natural Sciences and Psychology, Faculty of Science, Liverpool John Moores University, Liverpool L3 3AF, UK; K.B.Moejes@2016.ljmu.ac.uk (K.B.M.); s.t.durr@ljmu.ac.uk (S.D.); S.L.Conlan@ljmu.ac.uk (S.C.); 2 Faculty of Engineering and Technology, BEST Research Institute, Liverpool John Moores University, Liverpool L3 3AF, UK; reshmasulthana@hotmail.com; 3 Dept. Quality and Processing, Animalia AS, Norwegian Meat and Poultry Research Centre, P.O. Box 396 Økern, 0513 Oslo, Norway; alex.mason@animalia.no

**Keywords:** biological water pollution, *Tetraselmis suecica*, UV-Vis spectroscopy, low-frequency impedance analysis, electromagnetic wave sensors, algae detection

## Abstract

Biological water pollution, including organic pollutants and their possible transportation, via biofouling and ballast water, has the potential to cause severe economic and health impacts on society and environment. Current water pollution monitoring methods are limited by transportation of samples to the laboratory for analysis, which could take weeks. There is an urgent need for a water quality monitoring technique that generates real-time data. The study aims to assess the feasibility of three sensing techniques to detect and monitor the concentrations of the model species *Tetraselmis suecica* in real-time using eleven samples for each method. Results showed UV-Vis spectrophotometer detected increasing concentration of *Tetraselmis suecica* with *R*^2^ = 0.9627 and *R*^2^ = 0.9672, at 450 nm and 650 nm wavelengths, respectively. Secondly, low-frequency capacitance measurements showed a linear relationship with increasing concentration of *Tetraselmis suecica* at 150 Hz (*R*^2^ = 0.8463) and 180 Hz (*R*^2^ = 0.8391). Finally, a planar electromagnetic wave sensor measuring the reflected power S_11_ amplitude detected increasing cell density at 4 GHz (*R*^2^ = 0.8019).

## 1. Introduction

### 1.1. Pollution of Water with Microorganisms

Despite approximately 71% of Earth being covered with water, only 1% of that is freshwater accessible to humans [1,2]. An increasing global population and rise in associated anthropogenic pollutants, drive us towards the use of alternative water sources including seawater that can be treated and made potable [3]. Water quality is crucial to ensuring good health, ecosystem management, and economic development, and so it is imperative that we meet the increasing global demand for quality water. According to UN Water, the mortality rate due to poor water quality is nearly 3.5 million per year [4]. This has been addressed in The United Nations World Water Development Report 2015 and access to clean water has been named as one of the Sustainable Development Goals [5]. In order to achieve this goal, it is vital that various approaches are explored to ensure the standard of the water.

However, marine coastal waters are particularly vulnerable to pollution since coastlines are often highly urbanized with industrial activities and are considered the ultimate sink for sewage and other anthropogenic by-products [6]. Sea and brackish water organisms can cause significant levels of pollution themselves by either producing high concentrations of toxins harmful to other organisms, including humans, or by being a contaminant themselves [7,8,9]. Many of these pollutants have achieved worldwide dispersal or are reaching some non-native regions by hitching on ships as a transport vector [10,11]. This problem, resulting from ballast water [12] and biofouling on ship surfaces [13] is recognized by the international shipping community and international regulations are in place to limit transport and introduction of non-native species [14].

The pollutants in question are either marine larvae, algal spores or planktonic organisms in the water column (ballast water) [15] as well as disease organisms, all of which have the potential to cause serious harm to human health. An example of this is the recent ballast water induced outbreaks of Cholera due to the bacterium *Vibrio cholera* [16,17,18]. Some pollutants from ballast water are themselves not a direct risk to humans, but due to bioaccumulation, the toxins can cause serious illness in other organisms at higher trophic levels, including humans if the concentration is high enough [9]. Paralytic shellfish poisoning (PSP) is such an illness and is caused by a high concentration of saxitoxin due to blooms (red tides) of mostly single-celled planktonic algae (e.g., dinoflagellates) [19,20,21,22]. The incidence of the disease has increased over the last few years together with a greater number of red tides [23]. Algae releasing toxins will not only pose harm to humans via the consumption of food organisms, but also via waterways and freshwater production in desalination plants [9,24,25,26]. Therefore, it is important to develop a sensor that can detect potential harmful species and determine their concentration in aqueous environment.

### 1.2. State-of-the-Art Detection Technology

Several methods are currently used to detect and remove water pollutants. However, in this fast-paced world there is a need for a real-time analysis system to monitor water pollution in order to provide timely and informed decisions on remediation actions. This study aims to determine the feasibility of using optical, low-frequency capacitance, and low-power microwave measurement techniques for the real-time monitoring of living organisms that cause water pollution, using *Tetraselmis suecica* as a model species. *T. suecica* is a Chlorophyceae class green flagellate marine algae [27] found in many marine environments [28]. It is commonly used in aquaculture as feed for molluscs, crustaceans, and fish due to its relative ease of culture and high nutrient levels [29].

Electromagnetic wave sensors can be used in the detection of a broad range of substances including lactate [30], blood-glucose levels [31], and chemical compound concentrations [32]. In terms of water pollution, Huang, Wei, and Liao developed a biosensor using a recombinant gene of a non-pathogenic strain of *Escherichia coli* that changes color when in contact with arsenic, thereby providing information about the presence and concentration of the heavy metal [33]. In this fast-moving world, there is a need for such quick and cost-effective options readily employed in the field.

### 1.3. UV-Vis Spectroscopy

Optical spectroscopy is a well-established lab-based technique used for the detection of numerous water pollutants. It is a reliable standard, but it is bulky and may require additional reagents to produce a chemical reaction with an associated color change. It measures the absorbance, transmittance or reflectance values of a light beam that interacts with the sample under investigation. The interaction is dependent on the composition of the sample, hence allowing the user to distinguish between the components. Beer-Lambert’s law explains the relationship between absorbance and concentration in a simplified form:

(1) A=εlc

where *A* is the absorbance of the sample

*ε* = molar absorption coefficient (mol^−1^ dm^3^ cm^−1^)
*l* = path length of the sample (cm)
*c* = concentration of the sample (mol dm^−3^)

The above equation illustrates a linear relationship between the absorbance and the concentration of the sample. In this study, the same principle is applied to assess the relationship between absorbance and cell density of various *T. suecica* dilutions.

### 1.4. Low-Frequency Capacitance Measurements

A capacitance sensing structure with two cylindrical rods connected to an impedance analyzer can measure the dielectric properties of a sample, such as the impedance, when a potential is applied. The following formula can be used to calculate the capacitance of the sample:

(2) $$C=\frac{\pi \varepsilon_{0}\varepsilon_{r}l}{\ln\left(\frac{d}{a}\right)}$$

where *C* is the capacitance (F)

*ε*_0_ = dielectric constant of the sample between the sensor plates
*ε_r_* = permittivity of free space, 8.854 × 10^−12^ F/m
*l* = length of the rods (m)
*d* = distance between the rods (m)
*a* = radius of the rod (m)

The length of the rods, permittivity of free space, radius of the rod, and the distance between them remain constant, hence a linear relationship can be deduced between the capacitance of the sample and the dielectric constant of the same. The dielectric constant does not change for a particular sample, so if the capacitance is known, the dielectric constant can be calculated. This idea can be incorporated in a sensor to detect a living organism that may be present in a polluted water sample.

### 1.5. Low Power Microwave Sensing

Electromagnetic (EM) wave sensors operating at microwave frequencies are seeing an increasing interest across a variety of applications, including in the food industry and in water analysis [34,35,36,37,38,39,40,41]. The sensors can typically be characterized as requiring low power (<1 mW) while retaining a controlled level of penetration into a target material, in this case, polluted water. In this work, measurements from a planar type EM wave sensor were captured in the form of a reflected energy (S_11_). This energy is coupled into the sensor and the S_11_ signal varies depending on properties of the water sample presented to the sensing structure, such as conductivity and permittivity. Conductivity is a measure of a materials ability to conduct an electric current, whereas permittivity is a measure of how an electric field is affected by a dielectric medium. This is determined by the ability of a material to polarize in response to the field and reduces the total electric field inside the material. Therefore, permittivity (*ε_r_*), as defined in Equation (3), relates to a material’s ability to transmit an electric field and is a complex value which varies with frequency and accounts for both the energy stored by a material (*ε*′) as well as any losses of energy (*ε*″) that may occur.

(3) $$\varepsilon_{r}=\varepsilon^{\prime }+j\varepsilon^{\prime \prime }$$

The permittivity of a material is derived from a number of characteristics (e.g., temperature, chemical structure, molecular composition, etc.) and is a measure of various polarization phenomena that occur over different frequency ranges when exposed to an alternating EM field [42]. This leads to dipolar polarization in polar molecules (such as lactate), which causes them to rotate over a time period proportional to their dipole moment and local conditions (e.g., viscosity). Since there is a delay between the dipolar polarization and the applied alternating EM field, dispersions exists whereby the molecule does not have sufficient time to fully align to the field, giving rise to dielectric relaxation in the microwave region of the EM spectrum. A number of mathematical models have been developed by Cole and Cole [43], Cole and Davidson [44,45], and Havriliak and Negami [46] to explain relaxation phenomena. It is based upon these principles that EM wave sensors, operating at microwave frequencies, may selectively detect various species such as *T. suecica* in water.

## 2. Materials and Methods

### 2.1. T. suecica Culturing Process

Flasks of *T. suecica* were cultured in F/2 media and maintained at 18 °C with constant aeration and under artificial light [47,48].

### 2.2. Sample Preparation and Cell Count

A flask of cultured *T. suecica* was diluted with F/2 media to prepare the following concentrations up to 20 mL: 0%, 1%, 10%, 25%, 40%, 50% 60%, 75%, 80%, and 100%. To 1.5 mL of 100% *T. suecica*, 0.5 mL Lugol’s Iodine solution was added to immobilise the algae cells prior to counting. Cells were then visualised and counted on a haemocytometer at 10× magnification under a light microscope and cell density calculated. In addition to the 0% dilution (F/2 media only), 35 ppt enriched artificial seawater was prepared by dissolving Aquarium Systems Instant Ocean salt in distilled water and salinity was confirmed using a refractometer.

### 2.3. Optical Measurements with UV-Vis Spectroscopy

Three methods were implemented to compare the efficacy of their respective detection mechanisms. Using a spectrometer (Jenway 73 series), with a wavelength range from 200 nm to 1000 nm wavelength, the first parameter measured was absorbance. This method is widely used to measure algae biomass as the light absorbance can be directly related to cell density or cell number [49].

An absorbance baseline was first established using F/2 media and 35 ppt enriched artificial seawater (Aquarium Systems Instant Ocean salt, Blacksburg, VA, USA), followed by five repeats of each prepared sample in cuvettes across the wavelength range.

### 2.4. Low-Frequency Capacitance Measurements

**Figure 1:**
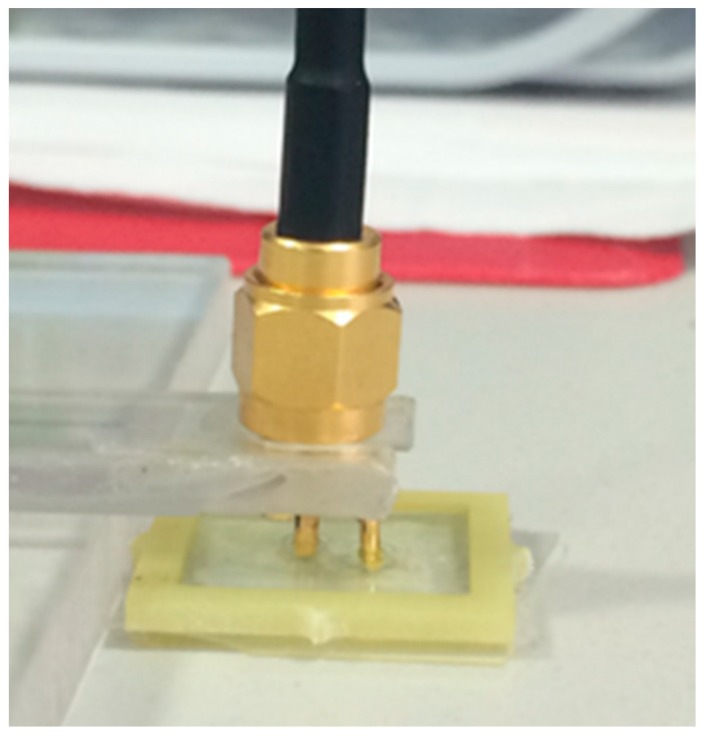
Gold plated sensor used in low-frequency capacitance measurements.

The second method considered was a Low-Frequency Impedance Analyser used to measure the capacitance of the *T. suecica* dilutions. A gold-plated sensor with two electrodes (Figure 1) was connected to a LCR Bridge (Hameg HM8118) and was used to detect the capacitance values for frequencies from 20 Hz to 200 kHz.

A purpose-designed holder seen in Figure 1 was filled with 400 µL of the sample, ensuring no air bubbles, and each sample had five repeat measurements.

### 2.5. Electromagnetic Wave Sensors

**Figure 2:**
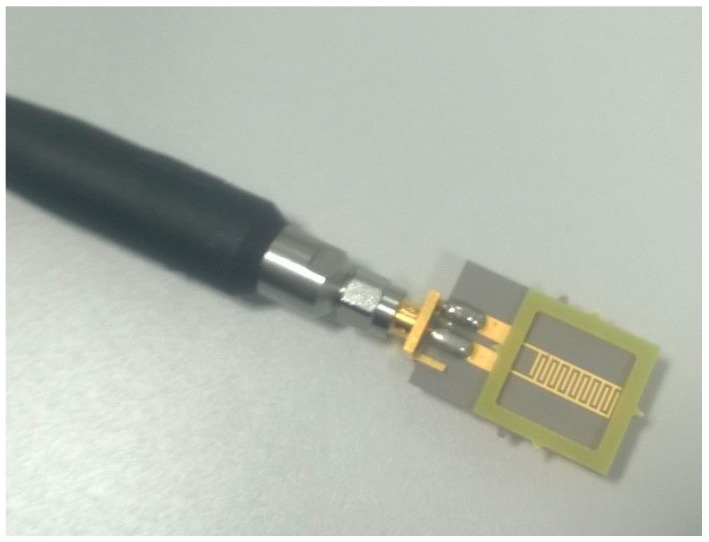
PTFE 8pr Au sensor with FR4 reservoir and SMA connector.

The third method examined was an electromagnetic wave sensor whereby the reflected power S_11_ was measured. A PTFE 8pr Au sensor (Figure 2) connected to a Vector Network Analyser (VNA, Rhode and Schwarz ZVA24) via one port was used to generate and receive a signal consisting of 60,000 data points from 10 MHz to 15 GHz. 400 µL of each sample was pipetted into the FR4 reservoir for measurements; this was repeated five times for each sample.

All experiments were undertaken in a temperature-controlled environment to minimize the effect of its variation on the experimental results, particularly due to the temperature dependence of the dielectric permittivity [50,51].

## 3. Results

### 3.1. UV-Vis Spectroscopy—Absorbance Versus Cell Density

**Figure 3:**
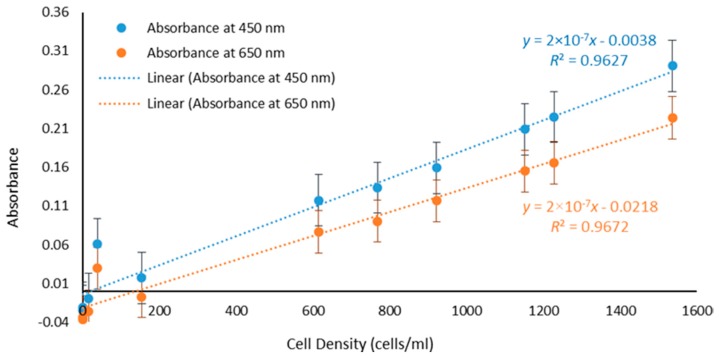
Linear increase in the value of the Absorbance with cell density at 450 nm (*R*^2^ = 0.9627) and 650 nm (*R*^2^ = 0.9672).

Absorbance values for the samples with different cell densities at 450 nm and 650 nm as well as F/2 only and artificial seawater are plotted in Figure 3. A statistical analysis reveals that the *R*^2^ values at 450 nm and 650 nm were 0.9627 and 0.9672, respectively. The data highlights the linear relationship between absorbance and cell density at a constant wavelength, concurring with Beer-Lambert’s law (1) and validating UV-Vis as an effective method for real-time assessment of algae biomass and water pollution. Absorbance can therefore be used as a parameter for measuring the presence of photosynthetic microorganisms.

Optical spectroscopy is an effective and well-established lab-based method for detection of photosynthetic living organisms. However, this approach is time consuming as the samples typically have to be brought back to the lab for testing and cannot be done in situ.

### 3.2. Low-Frequency Analysis: Capacitance Versus Cell Density

**Figure 4:**
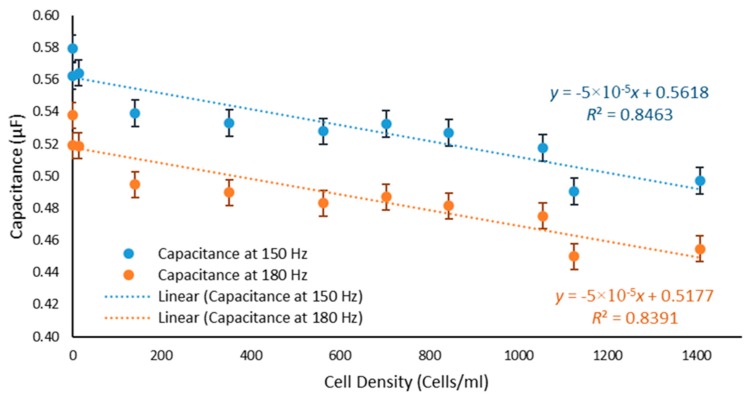
Dependence of capacitance on cell density.

Two frequencies, 150 Hz and 180 Hz, were selected and a graph showing a decrease in the value of the measured capacitance with increasing cell density is depicted in Figure 4. Statistical analysis of the data reveals that the *R*^2^ values for capacitance at 150 Hz and 180 Hz were 0.8463 and 0.8391, respectively. Furthermore, using the capacitance Equation (2) for a capacitor with two cylindrical rods, the dielectric constant of the material can be calculated. This can then be used as a database to detect the pollutant for a certain capacitance value.

### 3.3. Planar Electromagnetic Wave Sensors—Reflected Power S_11_ Versus Cell Density

**Figure 5:**
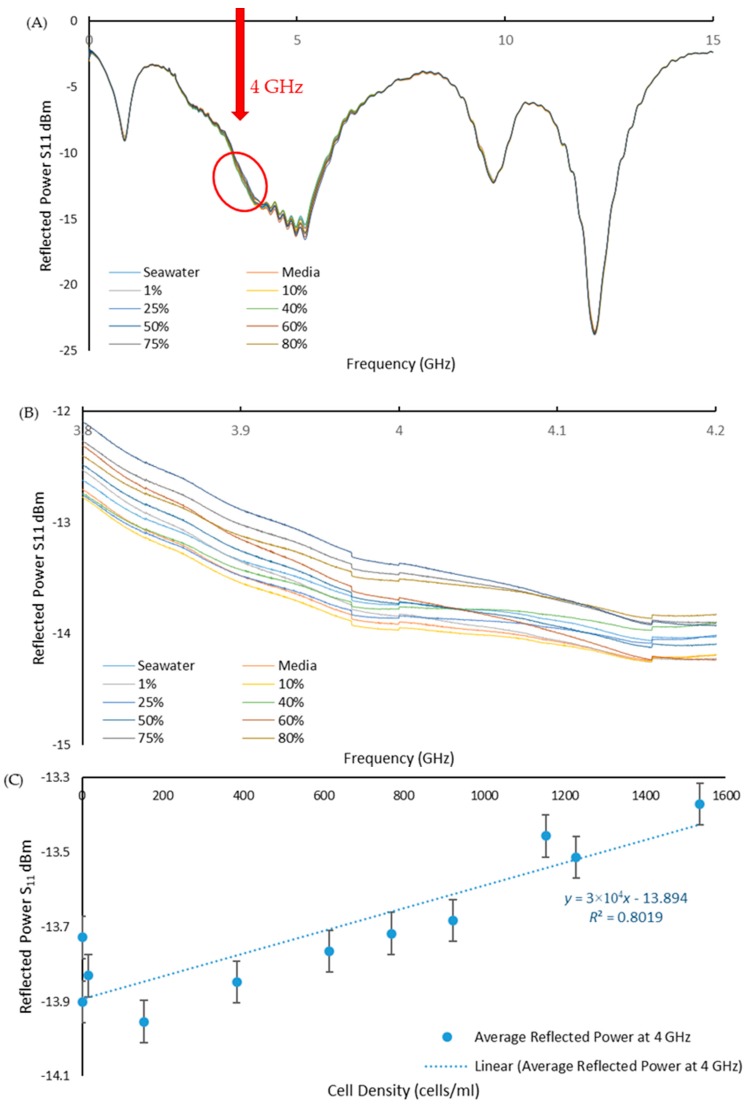
(**A**) Reflected power S_11_ versus frequency graph for PTFE 8pr Au in 0.01–15 GHz frequency range; (**B**) magnified part of spectra (**A**) (in red circle) in 3.8–4.2 GHz range to illustrate the effect of the cells concentration change on the amplitude of the reflected power signal; and (**C**) Linear change (*R*^2^ = 0.8019) in the reflected power S_11_ amplitude with increasing cell density at 4 GHz frequency.

The reflected power spectra were measured across the full frequency range to assess the sensors ability to detect the range of *T. suecica* concentrations (Figure 5A). Looking closer at around 4 GHz, Figure 5B shows the change in amplitude of the reflected power with cell concentration. As shown in Figure 5C, a selected frequency of 4 GHz provides a linear (*R*^2^ = 0.8019) calibration curve for change in the reflected power versus the cell density. This confirms that the planar electromagnetic wave sensor operating at microwave frequency range presents a viable option for real-time monitoring of water pollution with living organisms.

## 4. Discussion and Conclusions

To address the global need to develop a novel method for in situ water pollution analysis, three methods were investigated; absorbance measured with UV-Vis spectroscopy, low-frequency capacitance measurements on a LCR Bridge, and reflected power (S_11_) measured using a VNA with a low-power planar electromagnetic wave sensor. It was experimentally shown that these techniques were all able to detect the presence of the model species *T. suecica* at various concentrations.

UV-Vis spectroscopy demonstrates its well-established effectiveness as the experimental results verified the theory. At wavelengths from 400 nm to 450 nm and 650 nm to 700 nm the presence of various photosynthetic pigments, particularly chlorophyll *a*, allows for the detection of photosynthetic microorganisms [52,53]. It also validated Beer-Lambert’s law, showing a linear relationship between the absorbance and concentration with *R*^2^ values of 0.9672 for 650 nm and 0.9627 for 450 nm respectively.

The LCR Bridge showed an inverse dependence of the measured capacitance on the concentration of cells present in the samples, with *R*^2^ values of 0.8463 and 0.8391 at 150 Hz and 180 Hz, respectively. The capacitive change caused by the presence of algae has previously been shown [54]:

(4) $$\Delta C=C_{p,\mathrm{cell}}-C_{p,m}=\frac{1}{2\pi f}\times \left[\frac{Z_{\mathrm{mix},\mathrm{img}}}{{(Z_{\mathrm{mix},\mathrm{real}})}^{2}+{(Z_{\mathrm{mix},\mathrm{img}})}^{2}}-\frac{Z_{m,\mathrm{img}}}{{(Z_{m,\mathrm{real}})}^{2}+{(Z_{m,\mathrm{img}})}^{2}}\right]$$

where ΔC is the change in capacitance (F)

$C_{p,\mathrm{cell}}=$ Capacitance of the cell
$C_{p,m}=$ Capacitance of the medium
Z= Impedance
f= Frequency
$Z_{\mathrm{mix},\mathrm{real}}\;(Z_{m,\mathrm{real}})=$ Real parts of the complex permittivity of the suspension
$Z_{\mathrm{mix},\mathrm{img}}\;(Z_{m,\mathrm{img}})=$ Imagined parts of the complex permittivity of the suspension

The Equation (4) shows that capacitance will decrease with an increase in the measuring frequency as was shown when looking at 150 Hz compared to 180 Hz. Furthermore, with an increase in cell density, there is a decrease in the permittivity and therefore a decrease in capacitance.

Finally, a bespoke planar type electromagnetic sensor illustrated its ability to detect *T. suecica* at a range of concentration levels, with *R*^2^ = 0.8019 recorded for the reflected power S_11_ amplitude at around 4 GHz frequency, but non-linear dependence at other frequencies. At a cell concentration of zero, both the LCR Bridge and the planar electromagnetic sensor showed dependence due to the presence of salt in both the F/2 media and the enriched seawater medium being detected by the sensors as they also exhibit their own dielectric properties [55,56]. The decrease in relative permittivity with an increase in cell density results in a less negative reflection coefficient, as shown with the following equation:

(5) $$R=\frac{1-\sqrt{\varepsilon_{r}}}{1+\sqrt{\varepsilon_{r}}}$$

where *R* is the reflection coefficient.

$\varepsilon_{r}$ = Relative permittivity.

In conclusion, optical spectroscopy was the most established method, though the water samples would have to be brought back to the lab for analysis and would not be able to fulfil the requirement of the test in a small handheld device due to the need of minimal outside interference. However, there are several portable devices that are being developed that are less time-consuming, thus making them more suited for fieldwork. Notably, the bespoke microwave sensors offer a further practical solution for a cost-effective system for portable in situ evaluation of water pollution, instead of a VNA with 60,000 data outputs, only the minimum range of data points (frequencies) is needed for water pollutant detection and quantification in real-time. *T. suecica* is shown here as a model species to identify an alternative water pollution detection method using low frequency capacitance measurements or a planar EM wave sensor. These technologies can be further developed and applied for the detection of bio-fouling or invasive species in ballast water to desalination and water treatment plants. Nonetheless, it is necessary to identify key frequencies specific to a targeted species, which represents the largest challenge, but the authors are advancing the research in this area.
